# Supplementary material for: CD34T+ Humanized Mouse Model to Study Mucosal HIV-1 Transmission and Prevention
Source: Vaccines (Basel). 2021 Feb 27;9(3):198. doi: 10.3390/vaccines9030198 (PMC7997265; doi:10.3390/vaccines9030198)
Supplement: Supplementary file 1 [file vaccines-09-00198-s001.pdf]

Supplementary figures and tables

CD34T+ humanized mouse model to study mucosal HIV-1 transmission and prevention

Supplementary Figures

Supplementary Fig. 1 *Comparison of leukocyte populations in the blood of CD34 and CD34T+ mice*

Supplementary Fig. 2 *Non-humanized mice are not suitable for generating T+ mice*

Supplementary Fig. 3 *Comparison of leukocyte populations in the tissue of CD34 and CD34T+ mice*

Supplementary Fig. 4 *Sub-optimal mucosal HIV-1 infection in CD34 mice*

Supplementary Fig. 5 *Neutralization potency of bNAb tri-mix against NL4-3YU2 HIV-1*

Tables

Supplementary Table 1 *Leukocyte cell numbers in the blood of CD34T+ mice*

Supplementary Table 2 *Leukocyte cell numbers in the blood of CD34 mice*

Supplementary Table 3 *Leukocyte cell numbers in tissue of CD34T+ and CD34 mice*

Supplementary Figure 1

A

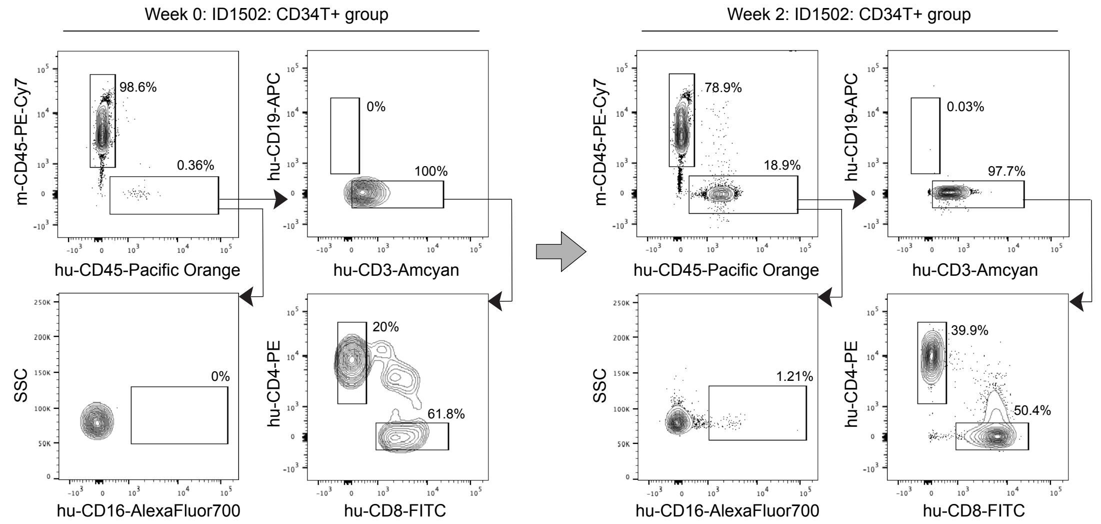

B

CD34 mice N=42			
Cell population	Mean cells/µl w0	Mean cells/µl w2	Fold expansion
hu-CD45+	95.9	83.0	0.9
hu-CD3+	21.3	24.1	1.1
hu-CD4+	11.9	15.3	1.3
hu-CD8+	5.7	7.2	1.3
hu-CD19+	67.8	51.5	0.8
hu-CD16+	1.0	1.8	1.7
mo-CD45+	818.1	827.7	1.0

CD34T+ mice N=69			
Cell population	Mean cells/µl w0	Mean cells/µl w2	Fold expansion
hu-CD45+	72.5	498.2	6.9
hu-CD3+	23.2	448.9	19.3
hu-CD4+	14.1	229.4	16.3
hu-CD8+	7.3	190.5	26.2
hu-CD19+	44.4	20.1	0.5
hu-CD16+	0.9	20.4	23.6
mo-CD45+	989.4	1243.6	1.3

Fold expansion
<5
5-15
>15

C

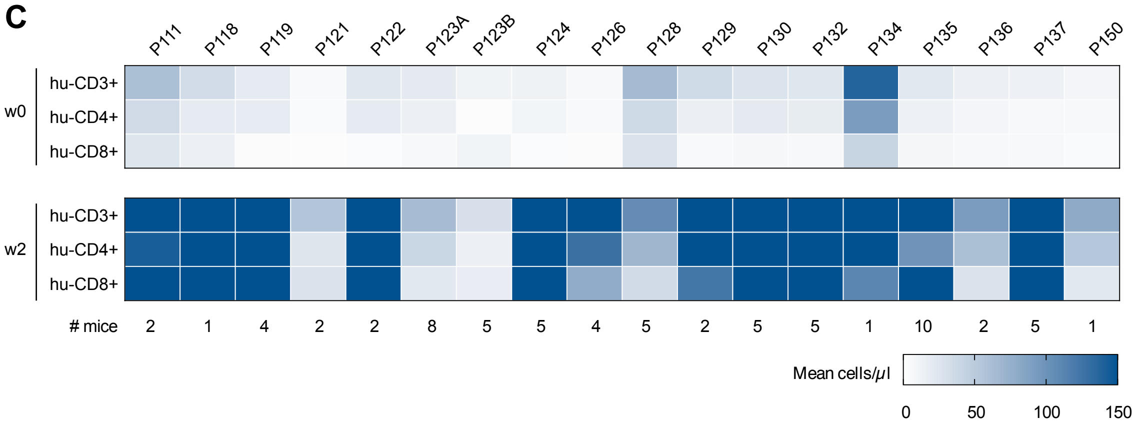

Supplementary Figure 1: Comparison of leukocyte populations in the blood of CD34 and CD34T+ mice. A, Gating strategy depicted with representative FACS plots from analysis of blood from a CD34T+ mouse ID1502 before (week 0) and after (week 2) UCBC and human IL-7 treatment. **B**, Mean cell numbers per µl blood and fold change between week 0 (w0) and week 2 (w2) from CD34 and CD34T+ mice shown in Fig. 1b. **C**, Analysis of human leukocyte expansion based on mean cell numbers per µl blood in CD34T+ mice (N=69) divided according to the cord blood donor.

Supplementary Figure 2

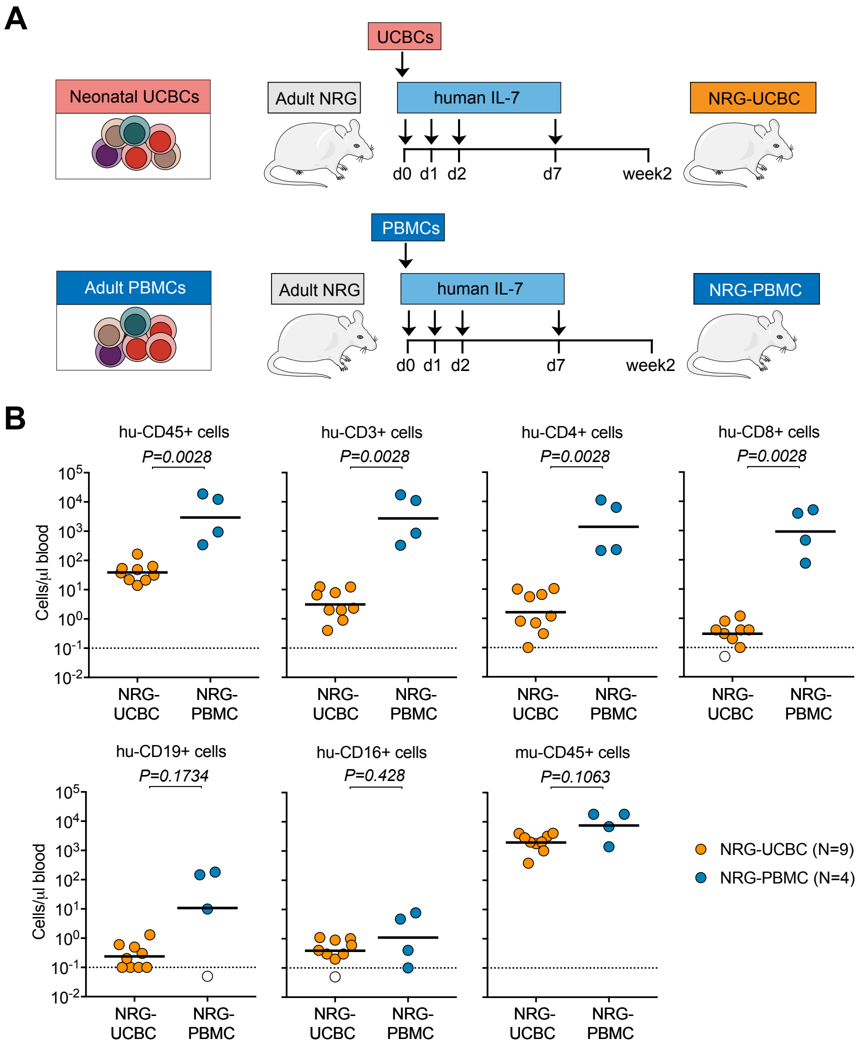

S2 Fig: Non-humanized mice are not suitable for generating T+ mice **A**, Schematic illustration depicting the generation of NRG-UCBC and NRG-PBMC mice. Non-humanized adult NRG mice were used for injection of either UCBCs or PBMCs along with human-IL-7. NRG-UCBC mice showed low human cell engraftment rates and NRG-PBMC mice developed rapid GvHD. **B**, FACS analysis of leukocyte populations in the blood of NRG-UCBC and NRG-PBMC mice, 2 weeks after injection of cells and IL7. Points shown in white were below limit of detection of assay. Statistical analysis done using unpaired Mann-Whitney test.

Supplementary Figure 3

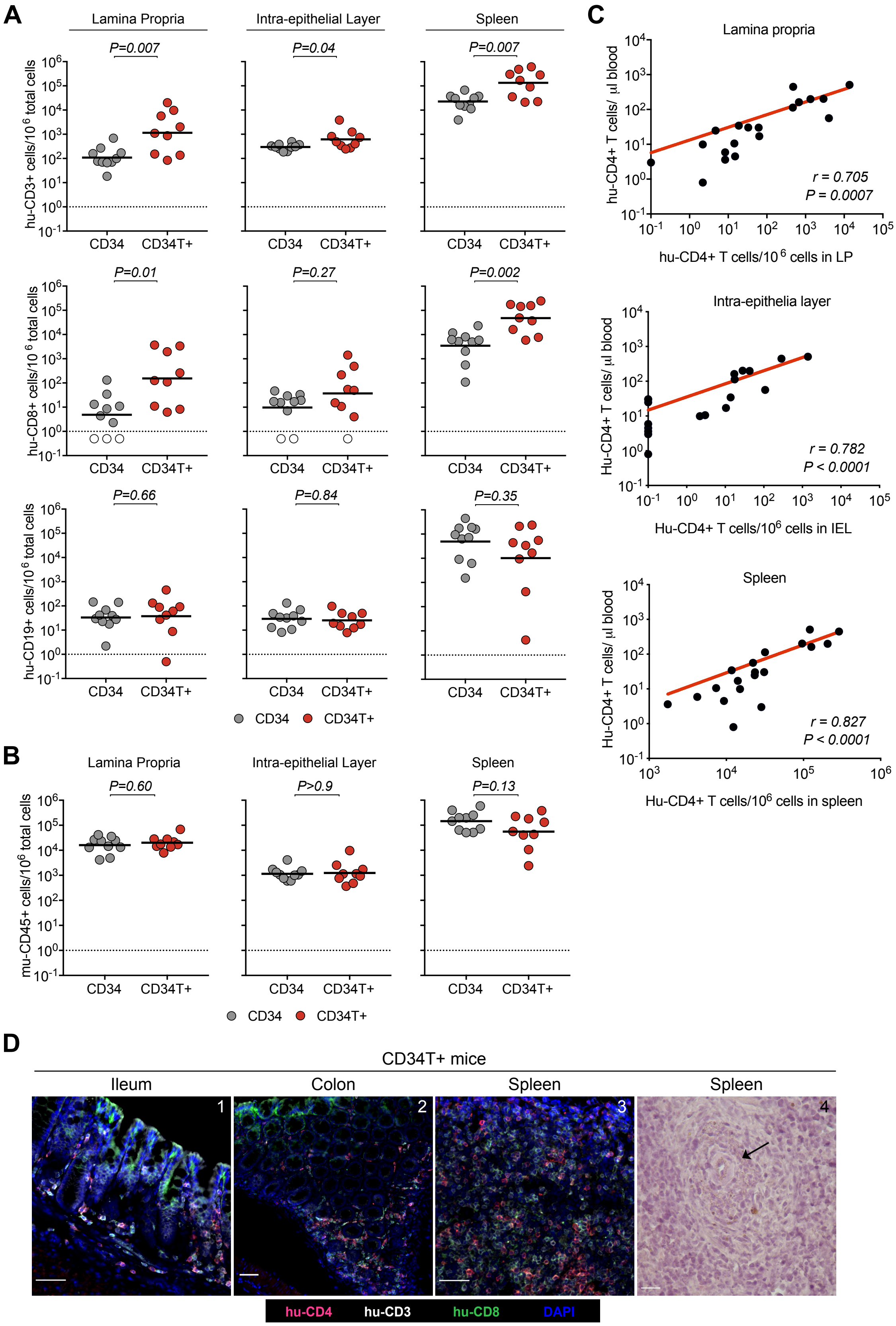

S3 Fig: Comparison of leukocyte populations in the tissue of CD34 and CD34T+ mice

FACS analysis of CD34 and CD34T+ mice shown in Fig. 2c for the presence of human CD3+ T cell, human CD8+ T cells, human CD19+ B cells (**A**) and mouse CD45+ cells (**B**) in the gut LP, IEL and the spleen at week 2. Points shown in white were below limit of detection of assay. Statistical analysis done using an Mann-Whitney U test. **C**, Combined Pearson correlation curves plotted for CD34 (N=10) and CD34T+(N=9) mice to analyze the relationship between human CD4+T cell levels in the blood vs. lymphoid tissue (LP, IEL and spleen). **A,B** Bars depict geometric means. **A,B,C** Dots represent individual mice.**D**, Image 1-3: IF staining of human T cells in the ileum, colon and spleen of HIV-1 infected CD34T+ mouse. Human CD3+ (white), CD4+ (red), CD8+ (green) cells were labeled for detection along with DAPI for visualizing the nucleus. Scale bar is 50 µm. Image 4: Section of splenic tissue from a CD34T+ mouse stained with Haematoxylin and Eosin demonstrating the presence of a PALS-like structure (arrow). 3 mice per group and 3 tissue sections from different areas of each mouse tissue were analyzed. Representative images were selected for display.

Supplementary Figure 4

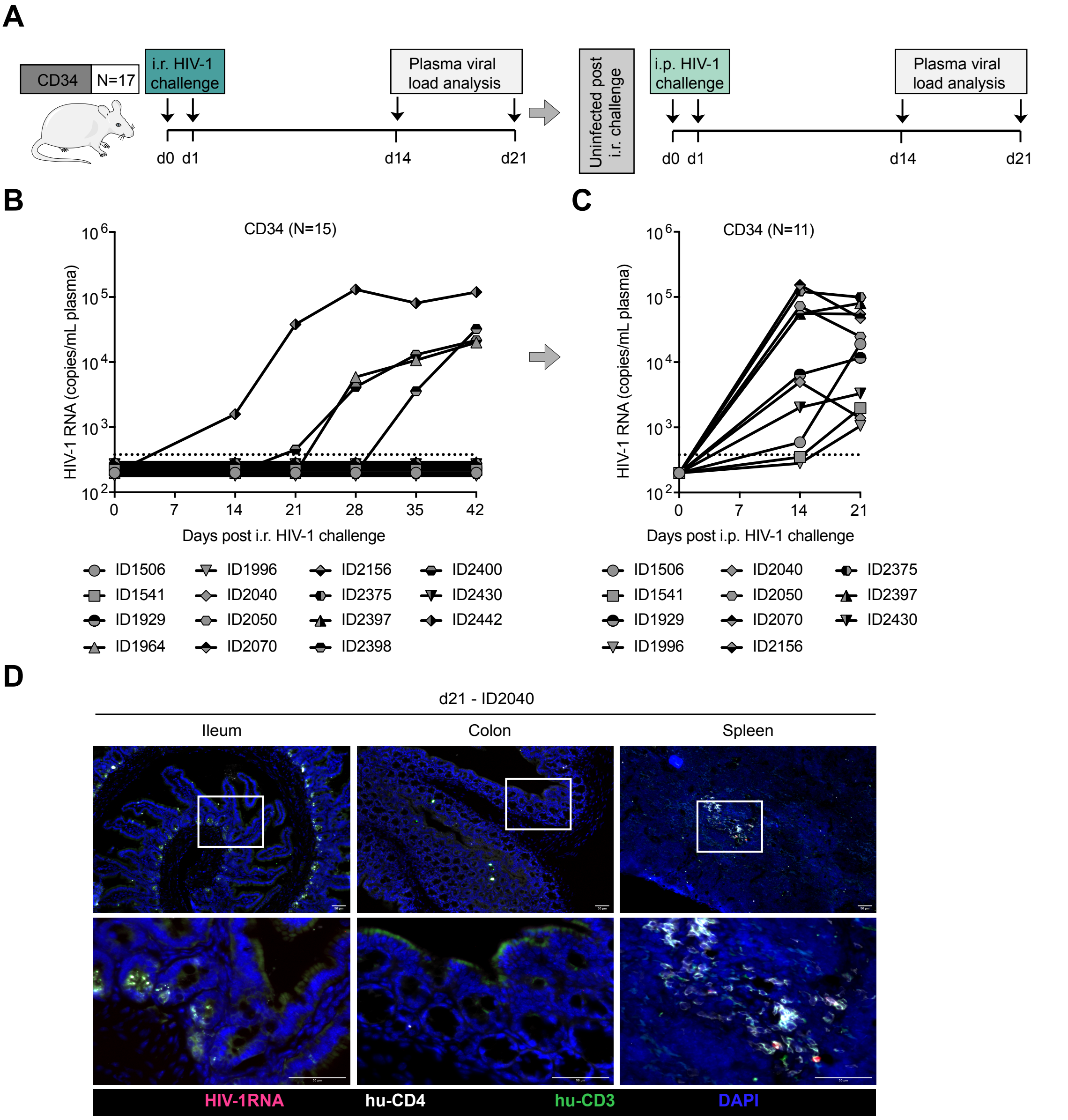

S4 Fig: Sub-optimal mucosal HIV-1 transmission in CD34 mice

A, Schematic representation of the experiment design. CD34 mice were given i.r. challenges with NL4-3YU2 HIV-1 on consecutive days and bled at the indicated time points for testing viremia. Mice that remained un-infected after i.r. challenge were challenged via i.p. injection of NL4-3YU2 HIV-1 to confirm susceptibility to infection. **B**, Plasma HIV-1 RNA levels in the blood of CD34 mice after i.r. challenge with high dose NL4-3YU2 HIV-1. **C**, Plasma HIV-1 RNA levels in CD34 mice after i.p. challenge with NL4-3YU2 HIV-1. The dotted line represents the detection limit of 384 HIV-1 RNA copies/ml of the assay in (b) and (c). **D**, RNA-scope-based detection of HIV-1 infection in the ileum, colon and spleen of CD34+ mice harvested at day 21 post i.p. HIV-1 challenge. HIV-1 RNA (pink), CD4+ cells (white), CD3+ cells (green) were labeled by in-situ hybridization and IF and nuclei were stained with DAPI. Scale bar is 50 μm. 3 mice per group and 3 tissue sections per mouse were analyzed. Representative images were selected for display. i.r. intra-rectal, i.p. intra-peritoneal

Supplementary Figure 5

A

		NL4-3$_{YU2}$ HIV-1		
Epitope	bNAb	IC50	IC80	MPI
V3 loop	10-1074	0.812	2.426	100
CD4bs	3BNC117	0.052	0.138	100
Silent face	SF12	0.914	2.456	100
Combi.	Tri-mix	0.107	0.320	100

IC50 (μg/ml)	< 0,5	0,5 - 2,5	2,5 - 10,0	>10
MPI	90-100	50-90	25-50	0-25

B

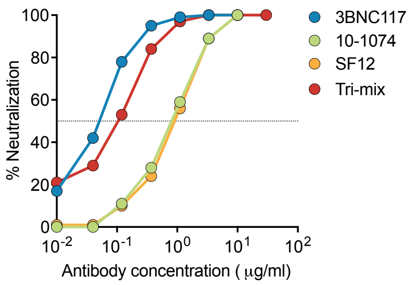

C

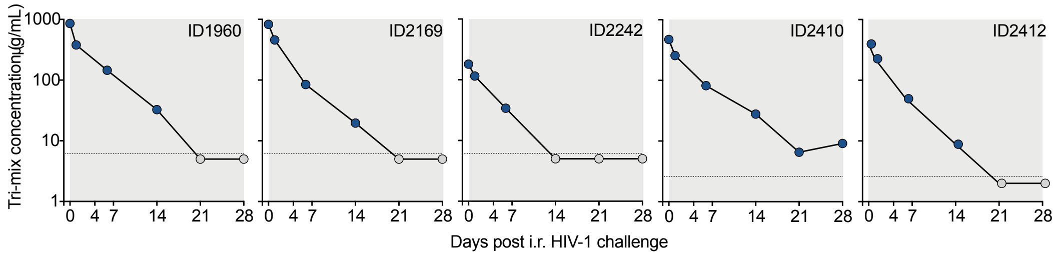

D

	Plasma ID50 antibody levels against NL4-3 YU2				
TP	ID1960	ID2242	ID2169	ID2410	ID2412
d0	6308	1330	6091	8155	6819
d1	2788	849	3361	4432	3901
d6	1068	250	622	1412	854
d14	240	45	144	480	152
d21	<45	<45	<45	113	<45
d28	<45	<45	<45	157	<45
d35	<45	<45	<45	<45	<45
d42	<45	<45	<45	56	<45
d55	<45	<45	<45	70	<45

E

	Plasma antibody levels in mice				
TP	ID1960	ID2242	ID2169	ID2410	ID2412
d0	857.89	180.88	828.38	472.99	395.5
d1	379.17	115.46	457.1	257.06	226.26
d6	145.25	34	84.59	81.9	49.53
d14	32.64	6.12	19.58	27.84	8.82
d21	6.12	6.12	6.12	6.55	2.61
d28	6.12	6.12	6.12	9.11	2.61
d35	6.12	6.12	6.12	2.61	2.61
d42	6.12	6.12	6.12	3.25	2.61
d55	6.12	6.12	6.12	4.06	2.61

S5 Fig: Neutralization potency of bNAb tri-mix against NL4-3YU2 HIV-1

A, Neutralization potency of individual bNAbs (3BNC117, 10-1074 and SF12) or an equimolar tri-mix (combi.) against the NL4-3YU2 HIV-1 infectious molecular clone represented as 50% inhibitory concentration (IC50) or 80% inhibitory concentration (IC80) along with the maximum percent inhibition (MPI) values. **B**, Neutralization curve of the individual bNAbs along with the tri-mix measured in (a). **C**, Antibody concentration of the tri-mix in the plasma of CD34T+ tri-mix mice from Fig. 3 measured using the TZM-bl assay. **D**, 50% Inhibitory dilution (ID50) values of the tri-mix in the plasma of CD34T+ tri-mix mice from Fig. 3 measured against the NL4-3YU2 HIV-1 infectious molecular clone. ID50 values at the time of i.r. HIV-1 challenge (d0 and d1) are highlighted in red. **E**, Tri-mix concentration in the plasma of CD34T+ tri-mix mice from Fig. 3 measured against the NL4-3YU2 HIV-1 infectious molecular clone. Values at the time of i.r. HIV-1 challenge (d0 and d1) are highlighted in red and values below the detection limit of the assay are highlighted in grey.

Supplementary Table 1. Leukocyte cell numbers in the blood of CD34T+ mice

CD34T+ mice N=69

Number of cells/µl blood volume: <10 | 10-100 | >100

Mouse ID	hu-CD45+		hu-CD3+		hu-CD4+		hu-CD8+		hu-CD19+		hu-CD16+		m-CD45+	
	w0	w2	w0	w2	w0	w2	w0	w2	w0	w2	w0	w2	w0	w2
**1317**	13,2	977,9	4,9	927,1	4,0	446,3	0,8	439,1	6,3	0,7	nd	nd	846,6	801,9
**1320**	41,3	1019,3	38,1	977,6	31,7	507,3	3,3	417,7	1,0	0,7	nd	nd	876,3	3291,4
**1344**	373,2	542,3	32,1	472,4	18,0	198,2	12,7	204,5	323,6	11,0	nd	nd	793,1	45,1
**1550**	98,1	632,7	54,8	615,6	32,7	202,0	19,1	354,6	38,4	9,2	nd	nd	719,4	201,3
**1571**	4,0	229,4	3,8	225,7	2,9	162,1	0,4	54,2	0,1	0,5	nd	nd	652,6	981,2
**1618**	0,9	22,2	0,7	21,5	0,1	9,9	0,5	8,8	0,0	0,1	nd	nd	673,3	911,8
**1800**	65,7	181,0	23,1	170,5	9,7	56,1	3,8	88,8	33,2	5,4	nd	nd	524,1	24,0
**1551**	78,5	260,0	62,9	256,2	31,9	79,8	27,1	156,8	10,6	1,7	nd	nd	538,6	219,3
**1578**	5,7	87,0	4,7	85,2	1,2	33,7	2,7	44,1	0,3	0,3	nd	nd	1874,5	1078,5
**1614**	32,2	140,4	31,9	139,1	26,3	87,9	4,2	45,4	0,0	0,0	nd	nd	1329,2	2288,0
**1782**	46,0	490,5	44,2	486,2	18,1	244,5	3,5	155,7	0,7	0,2	nd	nd	842,1	1930,3
**1502**	3,8	348,7	3,8	340,5	2,4	171,5	0,8	136,0	0,0	0,1	0,0	4,2	1052,2	1454,1
**1508**	63,8	141,0	63,2	139,7	59,4	100,0	2,9	32,7	0,0	0,1	0,1	1,2	1550,3	1322,3
**1529**	2,9	135,8	2,8	131,6	0,6	43,1	1,9	72,2	0,0	0,1	0,0	1,8	670,3	865,0
**1534**	2,1	1039,1	2,1	1014,1	1,4	406,3	0,7	470,8	0,0	0,4	0,0	23,0	1101,6	786,0
**1539**	0,5	260,9	0,4	256,1	0,1	135,3	0,2	109,3	0,0	0,0	0,0	2,8	510,3	2004,4
**1542**	12,5	289,6	12,5	282,8	8,8	174,9	3,5	99,1	0,0	0,2	0,0	4,4	1239,9	2861,7
**1901**	0,1	39,1	0,1	16,3	0,0	7,1	0,1	7,1	0,0	0,1	0,0	17,4	462,7	1184,8
**1915**	7,5	134,9	7,5	89,9	6,0	38,8	1,5	44,4	0,0	0,2	0,0	32,0	999,0	1774,1
**2021**	37,5	73,2	11,6	56,8	8,6	29,4	2,5	21,8	18,5	11,0	0,2	1,2	373,5	312,8
**2039**	13,2	97,2	0,6	94,2	0,3	33,8	0,1	44,7	10,6	0,4	0,1	1,8	553,4	418,4
**2047**	15,9	2065,4	9,6	2026,4	6,4	1197,6	2,1	723,4	1,8	0,0	0,9	32,2	301,3	1075,9
**1930**	16,8	64,7	6,5	23,1	5,4	17,6	0,9	3,6	7,4	29,7	0,3	5,3	342,3	1122,0
**1931**	50,8	164,6	29,7	117,9	22,3	81,3	6,3	31,8	14,1	35,6	2,2	7,1	547,8	764,7
**1956**	43,5	41,8	4,8	8,0	2,2	3,8	2,1	3,7	33,8	21,6	0,4	1,0	2004,9	10542
**1958**	36,9	44,8	10,1	19,7	7,3	13,4	2,2	5,0	22,4	19,9	0,8	2,3	1369,7	808,1
**1959**	50,0	127,2	12,8	45,8	9,2	32,7	2,5	10,8	29,3	54,7	3,7	20,1	657,2	812,5
**1960**	29,9	49,6	16,5	28,2	11,9	17,8	3,4	7,3	9,9	15,8	1,1	2,5	654,0	589,1
**1961**	60,8	139,2	36,4	121,3	19,8	65,3	14,6	48,6	14,6	7,3	5,2	10,1	287,3	95,6
**1962**	85,2	210,1	30,7	128,2	23,8	70,5	5,2	48,7	43,1	58,7	2,7	17,0	320,0	226,2
**2071**	43,7	43,3	42,8	42,4	0,1	13,8	39,6	26,4	0,0	0,0	0,1	0,1	3089,6	2544,1
**2098**	1,6	6,1	0,9	4,4	0,4	1,9	0,4	2,2	0,4	0,8	0,0	0,2	1153,0	1186,1
**2099**	0,9	15,3	0,9	14,8	0,9	10,4	0,0	4,1	0,0	0,0	0,0	0,1	1120,4	1237,4
**2109**	3,7	73,6	3,7	73,0	1,0	28,3	2,7	42,0	0,0	0,2	0,0	0,1	3764,3	5449,2
**2149**	102,4	288,0	32,2	204,6	16,0	105,7	15,3	88,7	57,7	63,6	1,3	7,5	702,7	690,1
**2150**	65,7	50,9	61,1	48,8	39,5	28,2	18,4	19,4	3,3	0,7	0,1	0,1	422,8	397,1
**2154**	258,6	199,6	176,1	179,2	90,3	149,9	82,1	26,7	73,3	16,6	1,2	0,5	986,5	768,7
**2168**	70,9	132,8	11,1	50,5	7,9	32,7	2,7	14,7	52,7	55,9	0,9	11,2	472,2	684,0
**2169**	31,6	37,5	30,8	37,0	18,6	20,6	11,3	14,7	0,2	0,1	0,1	0,1	528,0	797,8
**2242**	1,2	10,9	1,1	8,0	0,9	6,5	0,1	0,9	0,0	2,7	0,0	0,1	938,9	2610,6
**2372**	28,7	62,4	7,6	23,8	6,0	17,1	1,4	6,1	17,7	32,6	0,4	1,7	560,2	877,8
**2381**	211,7	305,5	3,8	54,2	1,2	34,4	2,5	11,0	191,6	201,5	1,4	29,5	1054,7	619,5
**2373**	51,2	1924,0	48,7	1906,8	30,2	1159,6	15,3	684,5	1,4	0,4	0,5	2,0	810,4	1742,6
**2376**	61,5	513,5	17,2	499,4	11,9	273,9	3,4	199,9	36,2	1,1	1,1	8,9	1263,2	542,6
**2405**	282,0	444,6	49,9	278,5	41,7	167,0	7,1	105,8	211,8	127,1	3,7	20,4	610,9	249,3
**2410**	55,2	146,0	54,1	143,0	41,1	35,8	8,6	101,6	0,9	0,0	0,0	0,2	1237,9	1215,2
**2412**	69,5	155,1	17,8	146,1	5,6	78,7	10,4	59,6	44,9	2,5	3,4	1,2	1307,0	963,7
**2415**	88,7	1101,7	81,3	1081,6	65,7	741,4	10,3	306,1	5,6	0,0	0,1	14,8	1907,1	607,1
**2417**	81,0	1762,5	9,5	1634,3	7,4	949,5	1,8	595,1	63,9	5,7	0,7	69,9	876,5	551,8
**2418**	159,1	4613,0	9,6	4093,2	7,0	2064,8	2,5	1769,1	136,3	29,8	0,5	338,3	1766,9	1445,9
**2419**	200,7	939,5	7,0	597,8	2,3	180,7	4,3	372,7	172,1	45,3	1,0	232,0	804,3	26,4
**2422**	79,3	1621,1	5,2	1489,8	2,5	892,5	2,6	476,9	70,5	3,2	0,7	91,0	1153,6	372,7
**2424**	52,1	238,4	19,2	176,6	13,8	73,2	5,2	85,3	30,4	44,3	0,7	14,4	1743,1	620,6
**2425**	32,6	301,6	5,4	281,9	3,4	95,4	1,3	157,8	24,9	5,6	0,2	8,7	882,2	949,5
**2426**	37,7	339,9	14,5	336,0	12,2	171,0	1,1	146,1	22,1	0,3	0,1	0,6	975,7	1846,7
**2429**	51,5	209,2	6,3	173,3	4,1	75,3	2,0	76,8	39,1	24,0	0,3	5,6	611,9	776,6
**2431**	91,2	787,3	23,6	755,1	10,5	244,8	9,6	462,9	58,5	4,4	1,2	14,1	1010,1	824,5
**2433**	285,0	384,4	6,7	246,8	1,0	75,0	2,8	138,3	262,1	75,5	2,2	49,7	722,5	94,5
**2434**	563,8	409,6	58,8	235,7	20,9	62,3	27,2	157,4	482,9	154,4	4,1	11,5	541,4	45,9
**2435**	60,8	322,2	5,4	263,0	3,1	64,7	2,0	182,5	50,2	49,2	0,4	6,0	1184,5	156,9
**2436**	35,8	230,0	2,9	221,1	1,6	53,9	1,0	128,7	29,9	3,5	0,2	1,7	1071,0	1344,2
**2438**	22,3	437,5	2,6	419,5	1,0	213,5	1,5	169,0	18,2	2,7	0,2	5,5	854,5	1775,7
**2439**	12,0	215,1	4,0	200,6	2,0	117,7	1,8	63,4	6,7	0,5	0,1	7,6	1854,1	993,7
**2443**	94,0	251,2	5,1	205,9	2,7	84,0	2,4	103,8	85,6	24,8	0,7	6,8	1361,4	841,0
**2444**	133,9	4889,2	44,0	4759,8	16,9	2260,5	16,9	2296,9	86,5	70,1	1,1	8,4	959,4	676,9
**2551**	118,1	172,1	7,4	78,1	4,3	52,1	2,9	20,2	104,8	71,7	1,1	15,8	914,2	672,9
**2571**	18,6	160,0	15,2	149,3	11,4	100,2	3,7	40,4	0,0	0,7	2,4	8,2	432,4	1202,3
**2575**	10,3	31,4	9,9	30,5	3,4	16,6	5,9	11,1	0,0	0,1	0,0	0,1	877,1	6681,7
**2649**	142,4	591,6	136,7	578,6	89,8	446,8	40,0	108,9	2,4	3,2	0,2	6,0	1077,0	1671,3

Supplementary Table 2. Leukocyte cell numbers in the blood of CD34 mice

CD34 mice N=42

Number of cells/µl blood volume: <10 | 10-100 | >100

	hu-CD45+		hu-CD3+		hu-CD4+		hu-CD8+		hu-CD19+		hu-CD16+		mu-CD45+	
Mouse ID	w0	w2	w0	w2	w0	w2	w0	w2	w0	w2	w0	w2	w0	w2
**1300**	447,3	258,2	4,5	7,1	12,2	24,8	17,4	33,5	408,2	193,4	nd	nd	641,4	255,0
**1306**	493,7	235,6	6,6	6,9	14,2	10,6	23,3	18,5	456,9	209,5	nd	nd	524,2	195,2
**1321**	42,2	134,3	5,7	10,6	24,2	113,0	31,0	124,9	9,9	0,4	nd	nd	803,2	773,2
**1337**	4,2	3,9	0,5	0,1	0,8	0,8	1,5	0,9	1,7	0,9	nd	nd	2340,8	3357,8
**1548**	45,2	54,6	23,8	21,5	19,3	30,3	44,9	54,4	0,1	0,0	nd	nd	804,5	591,7
**1576**	60,7	86,5	4,7	6,7	14,1	30,0	19,4	37,3	33,9	38,2	nd	nd	899,1	694,7
**1620**	7,0	17,1	0,0	0,6	1,6	3,0	1,7	3,9	4,5	11,5	nd	nd	945,5	936,8
**1802**	26,8	64,9	2,4	2,3	2,6	4,5	5,4	7,7	16,3	49,9	nd	nd	485,3	619,3
**1549**	115,6	161,9	14,8	24,9	27,0	50,3	43,7	78,4	64,6	71,6	nd	nd	427,0	382,9
**1617**	21,3	9,8	2,2	1,1	15,5	6,7	18,9	8,6	1,0	0,7	nd	nd	1809,4	840,0
**1619**	0,3	0,6	0,1	0,3	0,1	0,2	0,3	0,6	0,0	0,0	nd	nd	1300,3	1041,1
**1803**	213,3	38,6	36,9	9,4	57,5	13,9	210,2	38,2	0,7	0,1	nd	nd	381,1	1096,2
**1506**	1,9	1,5	0,4	0,5	1,3	0,6	1,8	1,4	0,0	0,0	0,0	0,1	956,8	935,7
**1535**	18,8	0,3	0,3	0,1	0,2	0,1	0,7	0,3	15,1	0,0	0,1	0,0	466,9	338,9
**1541**	3,3	2,1	1,5	0,9	1,2	0,8	3,3	1,8	0,0	0,0	0,0	0,0	838,2	648,9
**1996**	14,2	18,4	1,9	1,6	1,5	2,7	3,7	4,8	7,8	10,6	0,0	0,0	510,1	636,7
**2035**	18,6	7,3	3,0	0,7	5,9	0,9	8,9	1,7	7,5	4,8	0,0	0,0	1004,8	547,8
**2040**	23,2	15,2	0,4	2,7	13,4	11,1	14,7	14,6	7,6	0,5	0,1	0,1	247,8	695,7
**2044**	1,7	2,4	0,1	0,2	0,3	0,2	0,4	0,5	0,9	1,0	0,0	0,1	544,6	1150,6
**2048**	5,6	2,2	1,9	0,7	3,5	1,2	5,6	2,1	0,0	0,0	0,0	0,1	875,3	1518,0
**2049**	74,9	20,0	4,4	2,8	63,8	16,1	74,5	19,7	0,1	0,0	0,1	0,1	617,4	1724,6
**2050**	17,3	47,0	0,1	0,5	2,6	19,3	3,2	20,4	8,3	19,1	0,6	1,2	274,4	164,9
**1929**	78,5	120,0	10,2	10,6	16,6	32,7	29,0	45,3	37,3	63,8	1,6	5,2	400,2	416,9
**1964**	91,6	115,3	9,4	12,8	28,7	27,1	40,3	42,8	33,3	54,5	8,4	8,5	480,0	477,4
**2070**	23,0	16,8	0,2	0,0	22,6	16,4	22,9	16,5	0,0	0,0	0,0	0,0	1043,7	1917,9
**2151**	86,8	266,9	26,0	104,0	34,9	99,0	65,5	217,6	8,5	21,4	1,8	10,5	617,1	789,3
**2156**	12,1	11,0	3,2	3,8	8,4	6,3	12,1	10,8	0,0	0,1	0,0	0,1	983,1	814,6
**2171**	47,8	89,2	10,0	22,4	9,3	27,2	19,7	51,6	21,2	21,2	0,6	3,2	215,4	261,1
**2371**	54,6	60,6	2,2	2,1	3,4	3,6	6,3	5,9	42,7	48,8	0,3	0,3	1123,7	852,4
**2382**	115,0	112,9	5,1	7,6	2,9	5,9	9,2	14,6	90,6	80,0	1,4	2,3	612,7	473,3
**2375**	158,3	133,2	1,5	1,8	15,1	7,6	17,6	10,4	127,6	110,0	2,0	2,0	1263,5	742,7
**2378**	513,2	340,8	1,4	0,8	8,0	5,0	9,8	6,5	468,2	311,6	5,8	6,9	521,0	329,6
**2397**	297,6	308,7	3,9	3,9	3,7	6,0	9,0	11,2	264,7	272,6	1,7	4,5	864,1	858,5
**2398**	132,0	208,5	3,1	3,0	5,8	13,3	10,3	17,5	111,6	171,7	0,6	3,8	1215,0	876,2
**2400**	247,1	284,7	4,2	2,2	4,1	15,7	9,4	19,4	213,9	237,1	2,0	4,5	1180,1	693,8
**2421**	50,4	26,8	10,4	11,4	8,5	9,5	21,1	22,9	26,2	2,0	1,2	0,1	695,1	1313,5
**2430**	70,4	72,1	7,8	4,1	5,9	6,4	13,7	10,9	50,8	53,0	0,3	1,0	610,8	986,5
**2442**	234,5	33,9	11,1	6,6	14,8	10,0	27,0	18,8	189,3	13,3	1,4	0,3	739,4	1138,2
**2549**	47,4	31,2	2,0	2,1	11,2	10,1	13,6	12,2	29,6	17,8	0,3	0,1	556,2	660,9
**2550**	84,7	84,6	0,3	1,0	0,6	0,4	1,2	1,8	78,6	79,1	0,9	1,0	1022,1	665,6
**2573**	3,9	4,5	1,7	2,2	1,0	1,5	2,8	4,3	0,6	0,0	0,0	0,0	1083,7	1105,9
**2647**	22,4	18,2	8,3	5,2	10,3	10,4	20,2	16,5	1,2	1,0	0,2	0,1	1433,4	501,7

Supplementary Table 3. Leukocyte cell numbers in tissue of CD34T+ and CD34 mice

		No. of cells/ 10^6 cells in lamina propria at w2				
Model	Mouse ID	hu-CD3+	hu-CD4+	hu-CD8+	hu-CD19+	mu-CD45+
CD34	1306	267.6	14.4	34.5	141.0	12975.0
CD34	1321	688.6	468.6	131.5	145.8	4915.8
CD34	1300	70.0	4.7	0.0	39.7	4167.8
CD34	1337	99.3	2.2	11.0	17.7	24507.5
CD34	1576	171.0	61.4	13.2	67.9	26189.6
CD34	1802	77.3	15.0	8.6	27.9	23331.0
CD34	1548	70.3	33.0	4.4	2.2	35584.3
CD34	1620	18.3	0.0	2.3	29.8	14469.8
CD34	2371	159.4	8.3	0.0	22.8	41694.4
CD34	2382	66.8	8.3	0.0	41.7	13228.6
CD34T+	1344	2003.4	1343.6	260.0	91.8	14545.6
CD34T+	1317	868.3	477.4	126.2	131.1	7876.1
CD34T+	1320	20039.9	13773.5	3346.8	453.1	68751.0
CD34T+	1550	5709.2	2911.0	1937.1	60.7	21496.3
CD34T+	1800	9681.3	4027.8	3662.0	27.8	17683.4
CD34T+	1571	1099.5	675.1	109.9	88.0	27717.9
CD34T+	1618	83.9	2.2	11.0	8.8	16987.1
CD34T+	2372	135.7	63.7	8.2	0.0	28134.8
CD34T+	2381	151.8	18.7	6.2	41.6	13640.2

		No. of cells/ 10^6 cells in intra-epithelium at w2				
Model	Mouse ID	hu-CD3+	hu-CD4+	hu-CD8+	hu-CD19+	mu-CD45+
CD34	1306	330.6	3.0	33.4	48.5	1052.5
CD34	1321	250.5	17.6	7.1	31.7	1710.9
CD34	1300	192.0	0.0	15.6	131.7	1049.5
CD34	1337	372.1	0.0	29.3	69.9	4124.7
CD34	1576	495.3	0.0	47.0	34.2	602.0
CD34	1802	296.8	0.0	17.2	38.7	600.0
CD34	1548	293.1	0.0	19.3	12.8	1320.2
CD34	1620	186.0	0.0	17.1	10.7	1490.0
CD34	2371	386.3	0.0	0.0	23.3	1001.9
CD34	2382	307.2	0.0	0.0	8.1	767.9
CD34T+	1344	271.1	42.9	54.2	49.9	368.7
CD34T+	1317	1249.4	286.5	217.0	35.8	1727.7
CD34T+	1320	3835.9	1390.1	1419.7	97.3	9749.5
CD34T+	1550	341.0	28.2	50.0	50.0	480.0
CD34T+	1800	813.0	108.0	489.1	14.8	2536.5
CD34T+	1571	403.7	17.2	10.7	19.3	1140.2
CD34T+	1618	244.8	2.2	15.2	17.3	1148.0
CD34T+	2372	490.0	10.4	0.0	12.5	942.4
CD34T+	2381	739.6	13.8	4.0	7.9	765.3

		No. of cells/ 10^6 cells in spleen at w2				
Model	Mouse ID	hu-CD3+	hu-CD4+	hu-CD8+	hu-CD19+	mu-CD45+
CD34	1306	17114.5	7374.7	8082.3	161409.3	71989.1
CD34	1321	37657.2	31768.0	4971.5	6183.5	196772.1
CD34	1300	37727.8	23376.3	11706.6	443152.9	51384.4
CD34	1337	14563.5	12353.1	109.0	9113.9	588010.9
CD34	1576	30366.1	23547.6	5864.8	62731.9	251566.1
CD34	1802	16274.3	9329.9	5225.0	175796.5	399541.0
CD34	1548	67005.2	30973.4	23273.6	1525.3	145455.2
CD34	1620	31461.8	28440.5	555.1	71886.5	199539.6
CD34	2371	3950.1	1743.0	2049.7	94541.7	64010.2
CD34	2382	11367.4	4194.6	6204.8	187007.0	51005.0
CD34T+	1344	484012.0	205888.7	175039.3	210726.7	10652.7
CD34T+	1317	616356.2	289125.8	246492.7	34099.6	181182.1
CD34T+	1320	305262.1	120994.2	145051.5	417.8	131654.6
CD34T+	1550	285604.5	96094.8	157161.2	43804.5	55971.5
CD34T+	1800	90674.1	22217.6	49175.3	9393.8	2396.4
CD34T+	1571	169227.4	126501.9	37091.7	16658.4	224019.2
CD34T+	1618	35639.5	15070.2	16173.8	4.2	380874.6
CD34T+	2372	22477.2	14118.3	7700.5	55236.5	43175.4
CD34T+	2381	21337.2	11751.2	5853.4	236240.1	40745.5
